# Supplementary figures and images for: Test-retest reproducibility of in vivo oscillating gradient and microscopic anisotropy diffusion MRI in mice at 9.4 Tesla
Source: PLoS One. 2021 Nov 5;16(11):e0255711. doi: 10.1371/journal.pone.0255711 (PMC8570471; doi:10.1371/journal.pone.0255711)

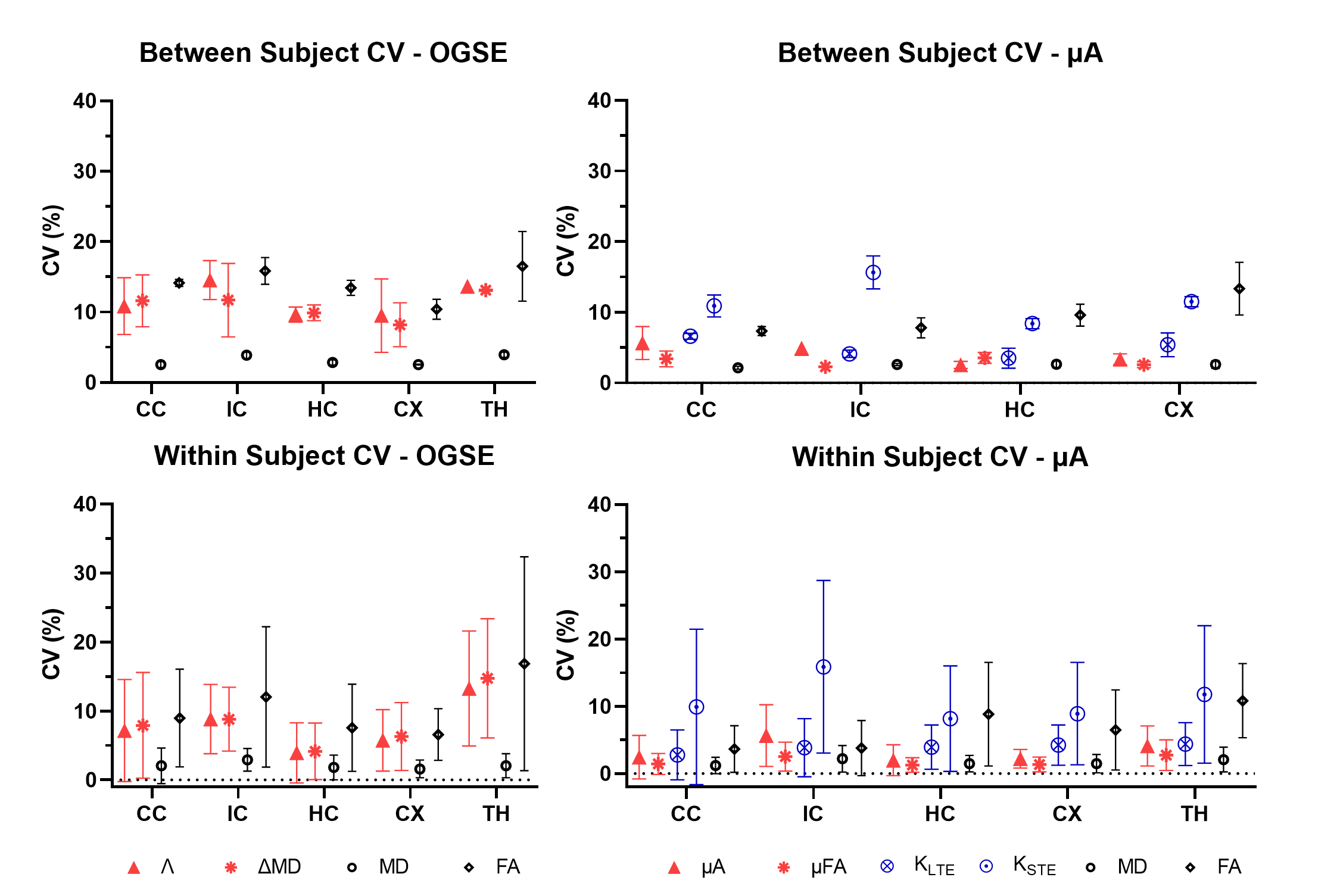

Supplement: S1 Fig — DTI metrics, MD and FA, acquired from both the OGSE and μA protocols, are shown as a reference. Values for the between subject condition represent the mean ± standard deviation over subjects (averaged over the test and retest timepoints). Values for the within subject condition represent the mean ± standard deviation between test and retest (averaged over the eight subjects). ROIs are abbreviated as follows: CC—corpus callosum; IC—internal capsule; HC—hippocampus; CX—cortex; TH—thalamus. (TIF) [file pone.0255711.s001.tif]

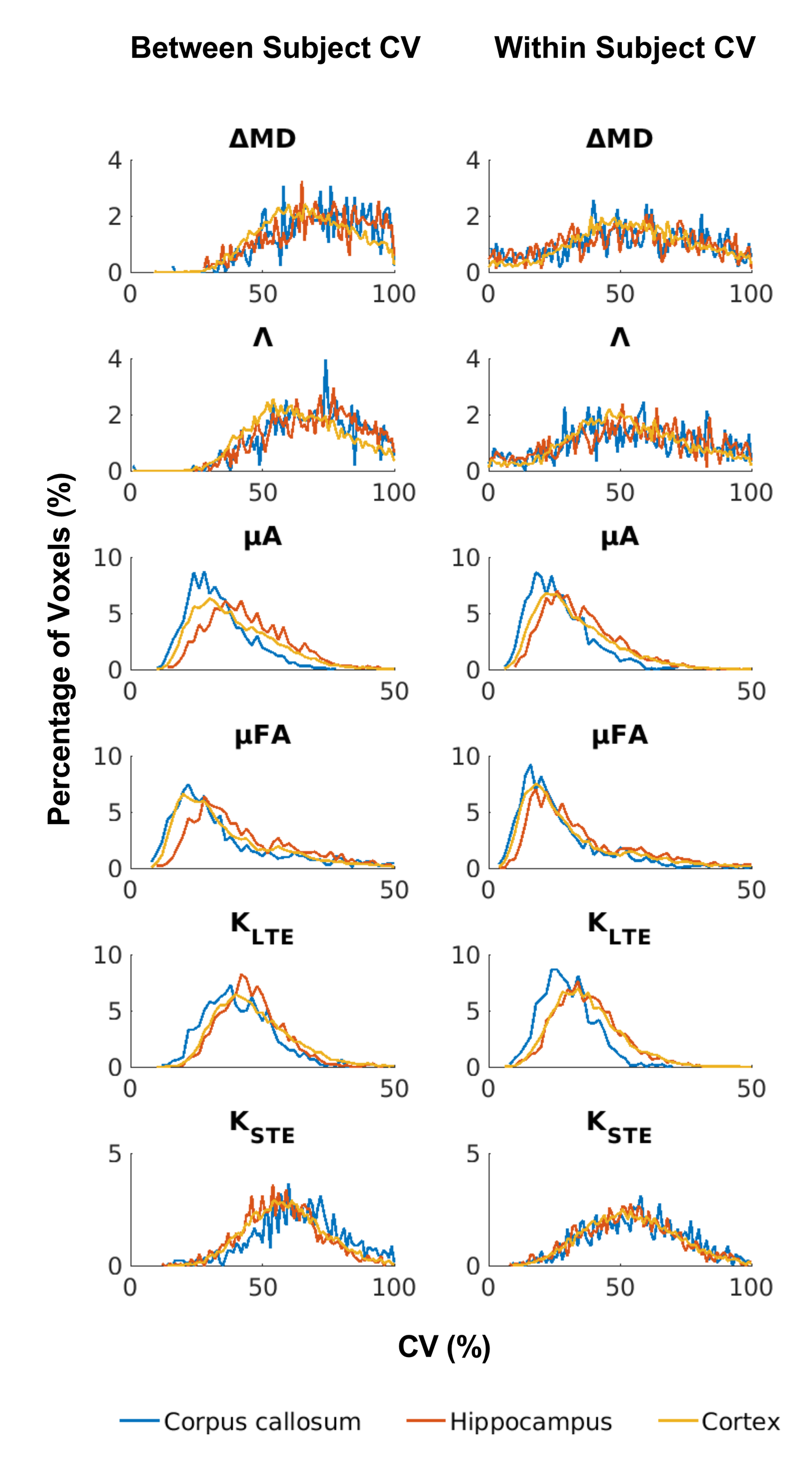

Supplement: S2 Fig — (TIF) [file pone.0255711.s002.tif]
